# Medical Student Engagement and Intention to Practise Medicine Across Undergraduate Years: A Longitudinal Study

**DOI:** 10.5334/pme.2157

**Published:** 2026-10-06

**Authors:** Hongbin Wu, Zehua Shi, Jie Xia

**Affiliations:** 1 Institute of Medical Education, Peking University, Beijing, 100191, China; 2 School of Management, Beijing University of Chinese Medicine, Beijing, 100029, China; 3 Faculty of Education, The Open University of China, Beijing, 100039, China

## Abstract

**Introduction:**

Medical students’ declining intention to practise medicine threatens workforce sustainability. Addressing this issue is critical to alleviating workforce shortages and improving healthcare delivery. While previous studies have explored factors influencing students’ career intentions, the specific role of multidimensional student engagement, and whether its associations with intention vary across undergraduate years, has not been systematically examined.

**Method:**

We analysed a two-year (2020–2021) longitudinal dataset from the China Medical Student Survey using a cohort-sequential design. Five dimensions of engagement were measured: behavioural classroom, behavioural clinical, emotional, cognitive, and agentic engagement. Multilevel growth models were applied to examine changes in intention to practise medicine and year-specific engagement-intention associations. Bayesian Model Averaging (BMA) was employed to evaluate robustness under model uncertainty using Posterior Inclusion Probabilities (PIPs).

**Results:**

The findings indicated that intention to practise medicine declined through Year 4, with a slight attenuation of the decline in Year 5. Engagement-intention associations varied across years: behavioural classroom engagement showed its largest association in Year 2; behavioural clinical engagement was significant in Years 4–5; cognitive engagement increased across years; and agentic engagement was stronger in Years 4–5 than in earlier years. Emotional engagement was consistently associated with intention across Years 1–5 and accounted for the largest incremental explanatory power; BMA indicated strong evidence for inclusion of emotional and agentic engagement.

**Conclusion:**

Engagement is consistently associated with intention to practise medicine, but the salience of engagement dimensions shifts across undergraduate years. Educational strategies should be year-sensitive and developmentally strategic—supporting emotional and cognitive engagement throughout training and cultivating agentic engagement in early years.

## Introduction

The quantity of healthcare workers and their competence in delivering high-quality healthcare services are pivotal to achieving universal health coverage [1]. However, healthcare systems worldwide face a substantial shortage of healthcare professionals [2,3]. Medical student attrition is a widespread phenomenon, and intention to practise medicine is an important predictor [4,5]. Furthermore, entering medical practice without a clear intention may lead to suboptimal medical practices, posing a significant challenge to the healthcare system [6]. Medical education aims to supply competent individuals willing to serve in the health sector [7]. Nevertheless, an increasing number of young individuals are losing enthusiasm for studying medicine or pursuing medical careers [8,9]. Many medical students choose to leave the medical profession after graduation [10,11,12]. In China, official exit surveys indicate that 13.04% of medical graduates withdrew from the medical profession upon graduation in 2019 [13].

Medical students’ intention to practise medicine is, to some extent, a reflection of their commitment to the medical profession [14]. Professional commitment represents an individual’s attitude towards their profession, and it can be operationalized as reluctance to leave one’s professional role [15,16]. Medical students’ lack of intention to continue a medical career upon graduation may, in a sense, point to an underlying withdrawal from the medical profession [17]. According to the Theory of Planned Behaviour, intentions are the proximal precursor of actual behaviour: the stronger an individual’s intention to perform a behaviour, the higher the likelihood of its occurrence [18]. As demonstrated in Sheeran’s meta-analysis article, a person’s intention is the most important predictor of his/her behaviour [19]. Therefore, medical students’ lack of intention to practise medicine may be expressed as attrition from the medical career pathway (e.g., not entering medical practice after graduation) [20], corresponding to a decline in professional commitment. Numerous studies have investigated factors associated with student attrition, including individual-, micro-, and macro-level factors [5,21,22]. Student engagement has been identified as a core educational factor associated with student attrition [23].

Student engagement is central to theories of academic persistence and attrition. An influential account is the participation-identification model [24], which posits that participation in educational activities and experiences of success can foster a sense of belonging, value, and commitment [25]. Engagement is therefore often conceptualised as a multidimensional construct that captures students’ participation and learning processes across behavioural, emotional, cognitive, agentic, and sociocultural domains [26,27]. In undergraduate medical education, engagement is enacted across both classroom and clinical learning contexts. Empirically, engagement has been associated with student retention and may buffer the impact of burnout [26,28]. Complementing this, socialization perspectives on professional training (e.g., Weidman et al.’s framework) emphasize that sustained involvement in learning and professional communities, as well as interactions with peers and mentors, are linked to the development of professional commitment [23,29,30,31,32,33,34]. Together, these perspectives suggest that engagement may be associated with medical students’ intention to practise medicine, and that the strength of these associations may vary across training stages [23,35,36]. In addition, self-determination theory suggests that when learning environments support needs for competence, autonomy, and relatedness, students are more likely to sustain intrinsic motivation [37]; accordingly, engagement may relate to intention partly through motivational processes, which we consider as an interpretive lens rather than a tested mediating mechanism in this study.

Moreover, a developmental perspective suggests that commitment to a professional pathway is formed and revised over time as learners encounter changing educational contexts and role expectations [23]. In the trajectory of career development, adolescence and young adulthood constitute critical periods for vocational exploration and establishment, and these phases are temporally embedded within higher education [38]. Career decision-making is often iterative, with students revisiting and refining intentions as they transition across phases of training (e.g., from classroom-based learning to more immersive clinical experiences) [39]. The salience of different engagement dimensions may shift by stage: forms of engagement that are most consequential in pre-clinical years may differ from those that matter during clinical training [39,40]. These perspectives suggest potential stage-specific patterns in how engagement relates to the intention to practise medicine.

Previous studies have examined factors influencing students’ intention to practise medicine, encompassing demographic characteristics [41,42,43], educational experience [43,44,45,46], skills [45,47], and career-related factors [46,48]. Educational experience factors include elements related to student engagement, such as time invested in learning and training, as well as practical and clerkship experiences. However, previous studies have not systematically examined student engagement within a comprehensive framework. Furthermore, most previous studies have explored the association between student engagement and outcomes such as academic achievement, well-being, and satisfaction [24,26,35], overlooking relationships with students’ intention to practise medicine. One previous study explored the trajectory of medical students’ intentions to practise medicine [49], but did not further analyse this relationship. Moreover, existing research is predominantly based on cross-sectional data, whereas studies using large-scale longitudinal data are lacking. This deficiency in research data precludes investigating the dynamics of student engagement and intention, as well as their relationship, during undergraduate medical education. Additionally, most prior studies employed traditional model techniques that assume a single optimal model. However, in the presence of complex and interrelated predictors, model uncertainty becomes a critical concern. Accordingly, we used analytic strategies to assess the robustness of engagement–intention associations (see Statistical analysis).

To address these content and longitudinal evidence gaps, our study examined how multidimensional student engagement relates to intention to practise medicine over time and across training stages. We posed the following research questions and hypotheses:

RQ1: How does medical students’ intention to practise medicine change during their undergraduate medical education?

RQ2: How does medical students’ intention to practise medicine relate to student engagement, and do these associations vary across years/training stages?

According to the Participation-Identification Model [25], students’ behavioural and emotional engagement in academic and clinical contexts is theorized to co-occur with stronger feelings of belonging and commitment to the learning community. Consistent with our framing, we use the Participation-Identification model to drive the expected direction of the engagement–intention association, without positing or testing an unmeasured mediating mechanism. In addition, socialization perspectives on professional training emphasise that sustained involvement in learning and professional communities, together with interactions with peers and mentors, is linked to the development of professional commitment [23]. Taken together, these perspectives support the expectation that higher engagement will be associated with stronger intention to practise medicine, and that such associations may vary across stages of training.

Guided by these perspectives, we propose Hypothesis 1: The more engagement, the higher the intention to practise medicine.

RQ3: Which dimensions of engagement show the most robust associations with the intention to practise medicine?

In parallel, the Self-Determination Theory provides a psychological mechanism that sustains career motivation through engagement [37]. Specifically, different engagement dimensions correspond to the satisfaction of three fundamental psychological needs—autonomy, competence, and relatedness. For example, agentic engagement reflects autonomy, cognitive engagement is linked to competence, and emotional engagement relates to the sense of relatedness. When these needs are met, students are more likely to develop intrinsic motivation and have the intention to practise medicine.

Based on this theoretical perspective, we propose Hypothesis 2: Emotional, cognitive, and agentic engagement are positively associated with medical students’ intention to practise medicine.

## Methods

### Context

In China, medical education begins at the undergraduate level. High school graduates who wish to become healthcare professionals are admitted to medical training programs based on their National College Entrance Examination scores, academic majors, and the medical schools’ admission quotas [50,51]. Students enrolled in medical training programmes in China spend five years completing general education, basic medical education, clinical medical education, and clerkship. The curriculum typically progresses from predominantly classroom-based learning in the pre-clinical years to more immersive clinical training and clerkships in the senior years. Medical training in China was recognised by the World Federation for Medical Education in 2020 [52]. During undergraduate training, institutional mobility is limited: students generally cannot transfer between colleges, and fewer than 5% can change majors within a college subject to specific requirements [13]. In this context, those who ultimately leave the medical pathway typically do so after completing the undergraduate programme and receiving the bachelor’s degree. Upon completing the five-year undergraduate programme, graduates in China must undertake standardised residency training typically lasting three years, before they are eligible for independent medical practise.

### Data Collection

Data were obtained from the China Medical Student Survey (CMSS) conducted between 2020 and 2021. The CMSS is a nationally coordinated, large-scale, multi-institutional survey by the National Center for Health Professions Education Development in China [53,54]. The CMSS collects information on basic demographic characteristics, pre-university experiences, student engagement, academic learning, extracurricular activities, academic performance, and career intentions of medical students in five-year programs. The CMSS is conducted annually in June, at the end of the academic year, and is the largest and most detailed survey of undergraduate medical education in China [36].

### Cohort Matching and Analytical Sample

The CMSS does not follow up on individual medical students; rather, it comprehensively assesses the quality of undergraduate medical education and the status of medical students from their perspective. However, as students’ unique IDs and college information are collected in each annual survey, individuals can be matched across survey years, enabling a longitudinal dataset [49]. We matched students’ unique IDs across 2020 and 2021 to construct a longitudinal, cohort-sequential dataset. Cohorts 1–4 comprised 20,264, 19,911, 15,885, and 11,379 students, respectively (Figure 1), with academic year advancing by one for each cohort (e.g., first-year students in 2020 became second-year students in 2021). The attrition rates for these cohorts were 44.62% (16,324), 37.26% (11,826), 47.90% (14,602), and 44.46% (9,108), respectively. Overall, the analytical sample consisted of 67,439 students from 94 medical schools across diverse regions of China, representing 26.85% (67,439/251,168) of the medical students enrolled across all responding medical schools. To assess potential attrition-related bias, we compared the matched longitudinal sample with the full 2020 and 2021 cross-sectional datasets on key demographic and engagement-related variables. No significant differences were observed, suggesting limited bias due to linkage attrition.

**Figure 1:**
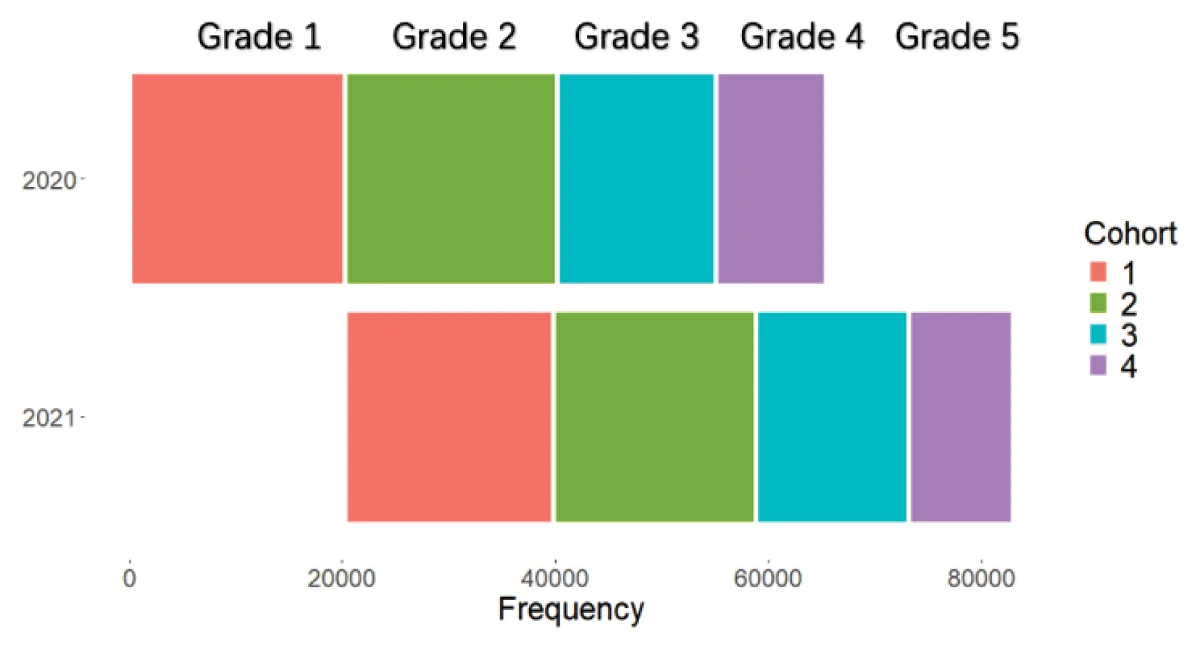
Data for the four CMSS cohorts used in this study.

### Variables

Intention to practise medicine was the dependent variable and was measured using a single item: *I intend to practise medicine after graduation*, rated on a 5-point Likert scale (1 = strongly disagree to 5 = strongly agree).

Student engagement was the independent variable. Based on the student engagement framework, we operationalised student engagement as a multidimensional construct encompassing behavioural, emotional, cognitive, and agentic components [36,55]. Behavioural engagement refers to positive conduct, persistence, effortful learning, and active participation; Emotional engagement refers to emotional reactions to learning; Cognitive engagement refers to psychological investment in learning and Agentic engagement refers to students’ proactive influence on their educational pathways to enrich their learning experience [26,36]. We excluded socio-cultural engagement primarily because we conceptualised it as the contextual layer embedded within each engagement dimension, and also because our study focused on a single national context [26,36]. Consistent with the structure of Chinese undergraduate medical education, we further separated behavioural engagement into classroom- and clinical-based engagement [35,36]. Accordingly, five dimensions of engagement were measured: behavioural clinical (3 items; B1-B3), behavioural classroom (2 items; B4-B5), emotional (3 items; E1-E3), cognitive (3 items; C1-C3), and agentic (3 items; A1-A3). The measurement properties and descriptive statistics of the engagement scales are presented in Table 1. Items used to investigate engagement were consistent with previous studies [35,55] using the same engagement framework [56,57,58,59]. Behavioural engagement items were measured on a 5-point frequency scale (1 = never to 5 = always), indicating how often students participated in each activity. Emotional, cognitive, and agentic engagement items were measured on a 5-point agreement scale (1 = strongly disagree to 5 = strongly agree).

**Table 1:** Measurement properties and descriptive statistics of student engagement scales.


	VARIABLES	MEAN	SD

	**Behavioural engagement:** Cronbach’s α: 0.78 (2020 wave); 0.75 (2021 wave) KMO:0.72 (2020 wave); 0.70 (2021 wave)		

	Behavioural clinical engagement		

**B1**	Participating in teaching rounds	3.71	1.13

**B2**	Participating in case-reporting	3.37	1.11

**B3**	Participating in clinical operations	3.21	1.15

	Behavioural classroom engagement		

**B4**	Doing academic-related presentations	2.80	1.02

**B5**	Participating in cooperative group learning or discussions	3.47	0.88

	**Emotional engagement:** Cronbach’s α: 0.89 (2020 wave); 0.79 (2021 wave) KMO:0.69 (2020 wave); 0.69 (2021 wave)		

**E1**	I am interested in my field of study	3.82	0.78

**E2**	I want my future career to be closely related to my profession	4.05	0.77

**E3**	I am passionate about learning	3.63	0.78

	**Cognitive engagement:** Cronbach’s α: 0.74 (2020 wave); 0.73 (2021 wave) KMO:0.70 (2020 wave); 0.72 (2021 wave)		

**C1**	When I encounter difficulties in my studies, I can usually think of some solutions to deal with them	3.79	0.73

**C2**	I have participated as an active learner with responsibility for my own learning	3.60	0.83

**C3**	I have assessed my competence	3.68	0.82

	**Agentic engagement:** Cronbach’s α: 0.78 (2020 wave); 0.75 (2021 wave) KMO:0.70 (2020 wave); 0.68 (2021 wave)		

**A1**	My feedback has been considered in curriculum development	3.51	0.90

**A2**	I have been involved formally and/or informally in peer teaching (explaining the appropriate knowledge to my peers)	3.60	0.86

**A3**	I have engaged in peer assessment	3.43	0.93


Notes: 1) This table presents the items used to measure student engagement; 2) the table shows the Cronbach’s alpha reliability coefficients and KMO (Kaiser-Meyer-Olkin) calculated for each engagement measure, separately for the 2020 and 2021 cohorts; 3) because behavioural clinical and classroom engagement are highly dependent on medical students’ period (the curriculum design in different grade), samples with an original behavioural clinical engagement’s and behavioural classroom engagement’s value of 0 were not included in the calculation for mean and SD; 4) Confirmatory Factor Analysis (CFA) was conducted to further validate the distinctiveness of the engagement dimensions. The CFA model fit indices showed a satisfactory fit: Comparative Fit Index (CFI) values ranged from 0.92 to 0.95 across the engagement dimensions, indicating a good model fit (CFI > 0.90). Composite Reliability (CR) values ranged from 0.75 to 0.88, supporting the reliability of the constructs. These results collectively confirm the validity and reliability of the engagement scales used in this study.

Based on prior literature and data availability, we adjusted for seven student characteristics that may relate to career intention: gender, place of residence (urban/rural), father’s education, mother’s education, father’s occupational status (ISEI), mother’s occupational status (ISEI), and having a medical worker in the family [53].

### Statistical Analyses

We assessed internal consistency of each engagement dimension using Cronbach’s α and examined the measurement structure of the engagement items using factor-analytic evidence.

To address RQ1, we summarised key variables using descriptive statistics and fitted multilevel growth models to characterise changes in intention to practise medicine across undergraduate years. These models are well-suited for analysing repeated observations of students nested in medical schools and allow for the inclusion of individual- and school-level variations [60,61]. As we examined two consecutive years, two-year data of each cohort were included (e.g., Cohort 1 provides the change trajectory from the first to the second year). We employed a cohort-sequential design to reconstruct the engagement trajectory across the full undergraduate period, yielding five observed time points spanning Years 1–5. A potential issue is the use of different cohorts to fit the trajectory. The existence of a common trajectory across all medical student cohorts must be demonstrated to ensure that trajectories do not vary by cohort. A naive analysis indicates no cohort differences if the (adjacent) cohort means are similar, and their confidence intervals overlap [62,63]. The results of this test are presented in the Appendix.

To address RQ2, we extended the multilevel growth model by incorporating the year variable, engagement, and the interaction term between engagement and academic year. We estimated models for each engagement dimension, then fitted a model that included all dimensions simultaneously to account for correlations across dimensions. All models were adjusted for seven covariate variables.

To address RQ3, we adopted Bayesian Model Averaging (BMA) to identify the key factors associated with students’ intention to practise medicine. BMA is particularly suitable in data-rich settings, as it accounts for the uncertainty of model selection by averaging over multiple possible models instead of relying on a single selected model. In our analysis, we included five engagement dimensions and seven covariate variables. The BMA algorithm applies Bayesian principles to update prior beliefs based on observed data and calculates a weighted-average coefficient (posterior mean) for each variable by accounting for its effect across all plausible models. Since our primary goal was to determine which variables matter, we focused on the Posterior Inclusion Probability (PIP) provided by the BMA algorithm—that is, the probability that a given variable has a non-zero effect. In essence, the PIP indicates the likelihood that each variable should be included in the “true” model, based on the combined evidence across all possible models. To interpret PIP, we used the following thresholds: <50% = no evidential effect, 50%–75% = weak evidential effect, 75%–95% = positive evidential effect, 95%–99% = strong evidential effect, and >99% = very strong evidential effect [64]. This approach helps distinguish between variables that are truly associated with the outcome and those that are likely to be noise, offering a more robust alternative to conventional regression methods that may overlook model uncertainty.

### Ethical Considerations

This study was approved by the Peking University Institutional Review Board (Ref. No. IRB00001052-20069) and was implemented in accordance with their guidelines and principles. The study objectives were explained to participants on the first page of the online survey. Students were able to proceed only after providing digital informed consent. Participation was voluntary, and confidentiality and the freedom to withdraw were guaranteed throughout data collection (participants could discontinue the survey at any time without penalty). For analysis, data were de-identified and reported in aggregate to protect participants’ privacy.

## Results

### Characteristics of the Sample Students

Analysis of student characteristics (Table 2) revealed that 41% of respondents were male, 54% were urban residents, and parental education averaged 10.51 years (fathers) and 9.44 years (mothers). Fathers also scored higher on the International Socio-Economic Index of Occupational Status (ISEI) than mothers. The mean value for having medical worker(s) in the family was low (0.11), indicating most students had no such occupational exposure.

**Table 2:** Student characteristics.


	VARIABLES	MEAN	SD

**1**	Gender (Male)	0.41	0.49

**2**	Residence (Urban area)	0.54	0.50

**3**	Father’s education duration	10.51	3.86

**4**	Mother’s education duration	9.44	4.32

**5**	Father’s occupation ISEI	33.04	18.89

**6**	Mother’s occupation ISEI	29.99	17.14

**7**	Having medical worker(s) in the family	0.11	0.31


Notes: ISEI: International Socio-Economic Index of Occupational Status.

### The trajectory of intention to practise medicine

As shown in Figure 2, students’ intention to practise medicine followed a distinctive arc across training years, declining from Year 1 through Year 4 and then largely stabilising in Year 5. Cohort-sequential consistency checks suggested minimal cohort differences in adjacent-year estimates (Appendix), supporting the interpretation of a common trajectory across cohorts. Figure 2b was based on an unadjusted model, whereas Table 3 (Model 1) reported covariate-adjusted estimates. In the adjusted model, the Year 5 coefficient was slightly less negative than for Year 4 (G4 = –0.183 vs. G5 = –0.176), indicating a small attenuation of the decline after adjustment.

**Figure 2:**
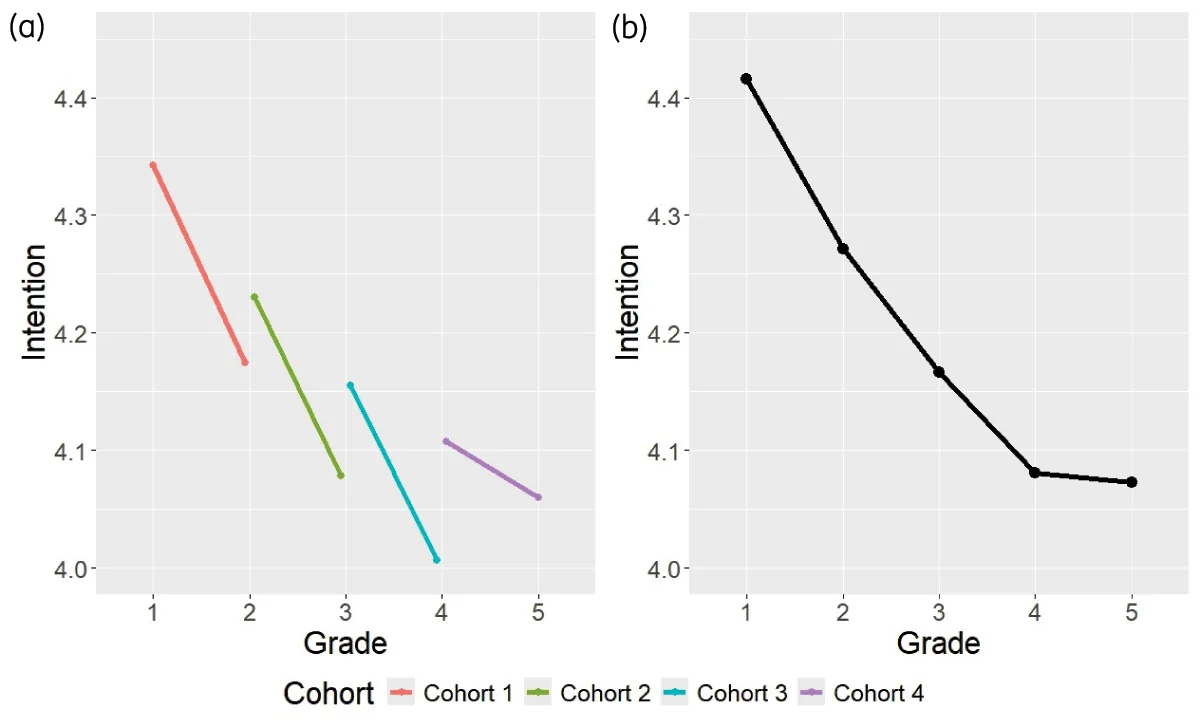
Students’ intention to practise medicine by year and cohort. **(a)** Descriptive mean intention by year for four cohorts; **(b)** Model-estimated trajectory from the multilevel growth model (without covariate adjustment).

**Table 3:** Regression results for the relationship between engagement and intention to practise medicine by year and cohort.


	MODEL 1	MODEL 2	MODEL 3	MODEL 4	MODEL 5	MODEL 6	MODEL 7

G2	–0.087***	–0.063* (0.029)	–0.109*** (0.027)	–0.089*** (0.005)	–0.099*** (0.005)	–0.089*** (0.005)	–0.079** (0.029)

G3	–0.138***	–0.108*** (0.028)	–0.123*** (0.026)	–0.128*** (0.006)	–0.145*** (0.006)	–0.131*** (0.006)	–0.096*** (0.027)

G4	–0.183***	–0.152*** (0.028)	–0.124*** (0.026)	–0.160*** (0.006)	–0.157*** (0.007)	–0.172*** (0.007)	–0.105*** (0.027)

G5	–0.176***	–0.203*** (0.028)	–0.127*** (0.026)	–0.176*** (0.007)	–0.198*** (0.008)	–0.180*** (0.008)	–0.155*** (0.027)

BEcli		0.016 (0.019)					–0.022 (0.018)

G2 × BEcli		0.051* (0.021)					0.034 (0.020)

G3 × BEcli		0.035 (0.020)					0.022 (0.019)

G4 × BEcli		0.096*** (0.019)					0.065*** (0.019)

G5 × BEcli		0.118*** (0.020)					0.064*** (0.019)

BEcla			0.050* (0.024)				–0.048 (0.025)

G2 × BEcla			0.048* (0.026)				0.039 (0.027)

G3 × BEcla			0.027 (0.025)				0.023 (0.025)

G4 × BEcla			0.035 (0.024)				0.039 (0.025)

G5 × BEcla			0.029 (0.024)				0.037 (0.025)

EE				0.336*** (0.005)			0.330*** (0.029)

G2 × EE				0.026*** (0.005)			0.006 (0.032)

G3 × EE				0.037*** (0.006)			0.035 (0.030)

G4 × EE				0.056*** (0.006)			0.027 (0.029)

G5 × EE				0.056*** (0.007)			0.036 (0.030)

CE					0.193*** (0.005)		0.065 (0.042)

G2 × CE					0.017** (0.005)		–0.027 (0.046)

G3 × CE					0.026*** (0.006)		–0.048 (0.043)

G4 × CE					0.042*** (0.006)		–0.043 (0.043)

G5 × CE					0.048*** (0.007)		–0.056 (0.043)

AE						0.140*** (0.005)	–0.022 (0.037)

G2 × AE						0.006 (0.006)	0.061 (0.041)

G3 × AE						0.007 (0.006)	0.067 (0.039)

G4 × AE						0.017** (0.006)	0.052 (0.038)

G5 × AE						0.030*** (0.008)	0.057 (0.038)

Student controls	Yes	Yes	Yes	Yes	Yes	Yes	Yes

School variance	0.005	0.007	0.006	0.001	0.003	0.004	0.002

Student variance	0.172	0.224	0.226	0.105	0.151	0.161	0.157

Student-year variance	0.338	0.298	0.298	0.303	0.320	0.329	0.262

School-level ICC	1.0%	1.3%	1.1%	0.2%	0.6%	0.8%	0.5%

Student-grade observations	134878	51614	51614	134878	134878	134878	51614

Num of students	67439	25807	25807	67439	67439	67439	25807

Num of schools	94	94	94	94	94	94	94


Note: 1) ICC, intraclass correlation coefficient (proportion of institution-level variance to total variance); 2) *p < 0.05, **p < 0.01, ***p < 0.001; 3) Student controls refer to the seven student characteristic variables listed in methods section; 4) G means Grade, G2 means Grade 2, the rest are similar; BEcli (Behavioural clinical engagement), BEcla (Behavioural classroom engagement), EE (Emotional engagement), CE (Cognitive engagement), AE (Agentic engagement); 5) Variance Inflation Factor (VIF) values were calculated to assess multicollinearity among the engagement dimensions included in Model 7. All VIF values were below the commonly used threshold of 10, indicating acceptable levels of multicollinearity. The VIF values were as follows: BEcli (Behavioural clinical engagement) = 1.271, BEcla (Behavioural classroom engagement) = 1.245, EE (Emotional engagement) = 1.955, CE (Cognitive engagement) = 3.073, AE (Agentic engagement) = 2.337; 6) To estimate the explanatory power of each engagement dimension, we calculated the marginal R² for each model (Models 2–6) and compared them with the baseline model (Model 1), which includes only control variables (grade and student-level covariates). The increase in R² after adding each engagement dimension reflects its individual contribution to explaining variance in medical students’ intention to practise medicine. The R² increments were as follows: EE (17.55%), CE (7.25%), AE (3.86%), BEcli (~0%), and BEcla (~0%). These values indicate that emotional engagement explains the most variance, followed by cognitive and agentic engagement, while the behavioural dimensions contribute minimally; 7) We also replaced the conventional linear model with Bayesian hierarchical modeling for this table, and the results remained broadly consistent.

### Engagement–intention associations and their variation across years

Table 3 (Models 2–7) reports year-specific associations between engagement dimensions and intention to practise medicine. In Model 2, behavioural clinical engagement was significantly associated with intention in Years 4 and 5. In Model 3, behavioural classroom engagement showed its largest association in Year 2, and no statistically significant differences were observed in other years relative to Year 1. In Models 4 and 5, the coefficients for emotional engagement and cognitive engagement increased across years. In Model 6, agentic engagement showed significantly stronger associations in Years 4 and 5, with no statistically significant differences from Years 1–3. In Model 7, which included all engagement dimensions simultaneously, behavioural clinical engagement remained significantly associated with intention in Years 4 and 5, and emotional engagement remained significantly associated with intention from Years 1 to 5.

We then compared the increments in R^2^ after adding each engagement dimension (Models 2–6). Emotional engagement accounted for 61% of the total incremental explanatory power across the five dimensions, followed by cognitive engagement (25%) and agentic engagement (13%), whereas behavioural clinical, and behavioural classroom engagement together accounted for <1%.

In addition, variance component estimates indicated that the largest variance component was at the student–year level, followed by the between-student component, whereas between-school variance was small. The school-level ICC ranged from 0.2% to 1.3% across models (Table 3).

### Engagement dimensions with the most robust associations

Table 4 presents the posterior inclusion probabilities (PIPs) from the Bayesian model averaging analysis. Emotional engagement and agentic engagement showed very strong evidence of association with intention to practise medicine (PIP > 0.99 for both). Behavioural clinical engagement also showed strong evidence (PIP = 0.99). Cognitive engagement showed positive evidence (PIP = 0.83), whereas behavioural classroom engagement showed no evidence supporting inclusion (PIP = 0.35). Figure 3 presents the posterior density distributions of the estimated coefficients for each engagement dimension. These densities provide complementary information to the Posterior Inclusion Probabilities (PIPs) reported in Table 4. Specifically, variables with high PIPs (e.g., EE, AE, and BEcli) exhibit posterior distributions that are tightly concentrated, indicating stable effects across model specifications. Notably, although the posterior distribution of BEcli is located close to zero in magnitude, it is narrowly concentrated and does not overlap with zero, suggesting a small but precisely estimated and consistently positive effect. In contrast, variables with lower PIPs (e.g., BEcla) display posterior densities that are more dispersed and centered around zero, suggesting weak and uncertain effects. Overall, this pattern highlights the consistency between model inclusion probabilities and the estimated effect distributions in the Bayesian model averaging framework.

**Table 4:** Bayesian model averaging estimates.


	PIP	POST MEAN	POST SD

Student Engagement			

EE	1.000	0.452	0.005

AE	1.000	0.048	0.006

BEcli	0.999	0.016	0.003

CE	0.828	0.017	0.009

BEcla	0.349	-0.004	0.005

Student Characteristics			

Mother’s education duration	0.776	0.003	0.002

Residence (Urban area)	0.381	0.009	0.013

Gender (Male)	0.052	0.001	0.003

Father’s education duration	0.026	0.000	0.000

Mother’s occupation ISEI	0.013	0.000	0.000

Having medical worker(s) in the family	0.011	0.000	0.002

Father’s occupation ISEI	0.007	0.000	0.000


Note: 1)PIP, posterior inclusion probability; 2)Variables are sorted by PIP values in engagement and student characteristics from highest to lowest. 0.000 indicates the numerical value being excessively small; 3)BEcli (Behavioural clinical engagement), BEcla (Behavioural classroom engagement), EE (Emotional engagement), CE (Cognitive engagement), AE (Agentic engagement); 4) Posterior Inclusion Probabilities (PIPs) for student engagement dimensions are analysed under three prior settings to assess robustness: (1) uniform (equal model probability), (2) fixed (constant model size), and (3) random (binomially distributed model size). PIPs remained consistent across priors, supporting the robustness of the findings.

**Figure 3:**
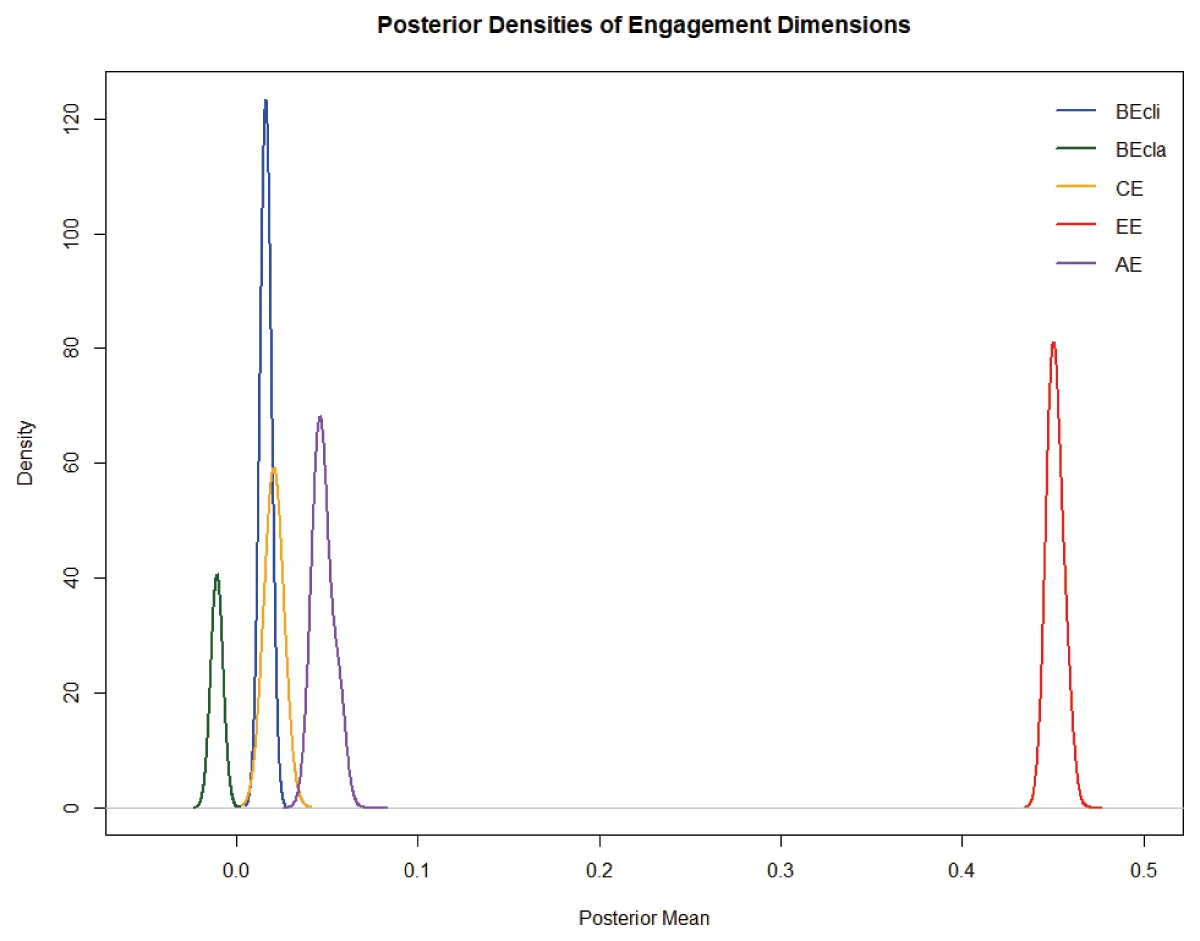
Posterior density plots.

## Discussion

This study found that medical students’ intention to practise medicine declined across undergraduate years through Year 4, with a slight attenuation of the decline in Year 5. We also found that multiple dimensions of student engagement were positively associated with intention. Although the strength of these associations varies across dimensions, this overall result provides empirical support for Hypothesis 1: The more engagement, the higher the intention to practise medicine. Compared with other dimensions of engagement, BMA results revealed that emotional and agentic engagement showed a very strong evidential support for inclusion in relation to intention, lending support to Hypothesis 2. These findings suggest that fostering engagement in various aspects of medical education may help strengthen students’ intention to practise medicine.

Importantly, the engagement–intention associations showed clear year-by-year variation, highlighting the “ebb and flow” across undergraduate training. In this study, behavioural clinical engagement was significantly associated with intention in the fourth and fifth years, whereas behavioural classroom engagement showed its largest association in the second year. Emotional engagement was consistently associated with intention from the first to the fifth year, and its coefficients increased across years. Cognitive engagement also showed increasing associations across years, and agentic engagement showed stronger associations in the fourth and fifth years than in earlier years. Against this backdrop, the largest decrease in intention was observed at the end of the first year, with a slight attenuation of the decline in the fifth year. In our prior work mapping student engagement trajectories in the same national survey, behavioural clinical engagement became relatively more prominent in the later years [65]. The late-year salience of behavioural clinical engagement may help contextualize the Year 5 pattern in intention. Furthermore, according to the socialization framework of Weidman et al., the development of students’ professional commitment is an upward-moving spiral process [23]. The Year 5 pattern is consistent with this developmental trajectory of professional commitment. Moreover, variation in intention appeared to stem primarily from individual-level differences associated with academic-year progression, whereas differences between schools were comparatively minimal. These findings align with those of a previous study [49]. All medical schools in China follow the Standards for Undergraduate Medical Education to ensure the homogenisation of professional training. This standardization may reduce inter-school variation in students’ intention to pursue a medical career, though other unmeasured institutional factors may still play a role [66].

The association between emotional engagement and intention was among the strongest in our findings. Emotional engagement appeared to become increasingly important with each academic year. This finding supports previous research findings indicating that emotional engagement may motivate students and shape their career intentions [36], for example, emotional experience affected students’ confidence in the medical profession [67]. Emotional engagement reflects perceptions of learning value [68]. Higher perceived value is associated with greater emotional engagement in learning. This fact is often associated with improved learning outcomes. The participation-identification model states that engagement in school activities and success experiences promotes students’ sense of belonging and value [25]. According to self-determination theory, satisfying students’ needs for competence, autonomy, and relatedness helps maintain intrinsic motivation. A previous study found that emotional engagement was significantly positively associated with medical students’ behavioural and cognitive engagement [35]. Consistent with the multidimensional nature of engagement, these correlations may help contextualize why emotional engagement accounted for the largest share of incremental explanatory power in our models.

Agentic engagement was positively associated with intention, particularly in the fourth and fifth years of undergraduate medical education. While previous studies have found that agentic engagement predicts learning achievement and student satisfaction [24,35], our findings suggest it may also contribute to students’ professional commitment in later years of undergraduate medical education. Agentic engagement reflects proactive behaviours and interactivity in the professional community and institution. Students may strengthen their intention to practise medicine through interacting with other professionals and participating more centrally in the learning community [69]. According to self-determination theory, agentic engagement may support students’ autonomy and sense of competence, which, in turn, enhance intrinsic motivation and facilitate the internalisation of professional values. Rather than directly determining outcomes, agentic engagement may help foster a motivationally supportive learning environment [70], thereby contributing to students’ sense of belonging and commitment. Notably, however, its incremental explanatory contribution was smaller than that of emotional (and cognitive) engagement in our analyses, suggesting that “robustness” and “magnitude of explained variance” may not fully coincide across dimensions.

To further contextualise these year-specific associations, in China’s five-year curriculum, earlier years are predominantly classroom-based and focus on foundational and biomedical learning, whereas later years involve more immersive clinical training and clerkships. Accordingly, behavioural classroom engagement showed its largest association with intention in Year 2, whereas behavioural clinical engagement was significantly associated with intention in the fourth and fifth years, when clerkships and rotations are typically scheduled. These findings are consistent with the idea that clinical learning experiences in later years may co-occur with a stronger intention to practise medicine, potentially reflecting students’ perceived competence and belonging in clinical teams [35,71], as suggested by self-determination theory and professional socialisation perspectives.

Finally, when the five dimensions of engagement were included in the regression analysis, only behavioural clinical and emotional engagement remained statistically significant. This may be because, in multivariable linear regression models with highly correlated variables, if a variable’s contribution has already been accounted for by other variables, that independent variable ceases to be significant, even if it originally showed a notable association with the dependent variable. In contrast, this study employed Bayesian model averaging, which assigns weights to multiple models to account for model uncertainty [72].

## Practical Implications

This study highlights the significant association between student engagement and medical students’ intention to practise medicine. Emotional engagement showed consistent associations across Years 1–5 and accounted for the largest incremental explanatory power in our models, whereas agentic engagement showed very strong evidence for inclusion and stronger associations in Years 4–5. These findings suggest that medical educators should prioritise strategies that support emotional engagement across the curriculum and enable agentic engagement, particularly in later stages of training, to help medical students solidify their intention to pursue the medical profession.

To cultivate emotional engagement, educators may consider enhancing the situational and experiential nature of courses, incorporating structured reflective learning activities, and creating opportunities to strengthen students’ emotional connection to clinical practice. Mentorship programmes or role model-based guidance can provide social and emotional support and reinforce students’ professional aspirations. To promote agentic engagement, students can be encouraged to participate in curriculum design, engage in peer teaching, and contribute to establishing fair and transparent assessment criteria. Faculty responsiveness to student feedback, recognition of initiative-taking behaviours, and implementation of supportive reward systems may further reinforce students’ sense of autonomy, competence, and belonging.

Our findings indicate that the strength of association between particular engagement dimensions and intention to practise medicine varies across training years, suggesting that interventions should be sensitive to developmental stage. However, this year-sensitivity need not imply rigidly compartmentalised, year-specific interventions; rather, it calls for a strategically scaffolded approach that deploys engagement-oriented interventions across the entire undergraduate trajectory. For example, although agentic engagement demonstrated its strongest association with intention in Years 4 and 5, promoting it earlier (during Years 1–3) could cultivate foundational proactive learning dispositions. Similarly, emotional and cognitive engagement may be fostered continuously throughout training. Thus, the key consideration is not merely when to intervene, but how to strategically sequence and adapt engagement-oriented interventions across dimensions and across time.

## Limitations

This study had several limitations. First, the data were collected from medical students in China. Although the study provides insight into intention in this context, the transferability of its findings to other educational systems should be approached cautiously. The structure of medical training varies across countries, which may affect how behavioural engagement is expressed and measured, and the meaning and expression of agentic engagement may differ across cultural contexts. For example, in China, undergraduate medical curricula may offer more limited opportunities for students to exercise agentic engagement [34]. Future research could examine engagement–intention associations in other cultural and educational settings using comparable measures.

Second, this study used longitudinal data to track changes in engagement and intention over a two-year period. A longer follow-up period would provide a more comprehensive understanding of how engagement and intention evolve, and how their associations may change over the course of medical education. In addition, the measurement of behavioural classroom engagement was limited to two items, which may have reduced estimation precision and contributed to its lower posterior inclusion probability. Third, although we adjusted for several individual characteristics, other potentially relevant factors (e.g., financial considerations, family expectations, and labour market conditions) were not included and should be explored in future research. Finally, as an observational study, the findings should not be interpreted as causal; future work could incorporate quasi-experimental designs or longitudinal interventions to strengthen causal inference regarding the engagement–intention relationship.

## Additional File

The additional file for this article can be found as follows:

10.5334/pme.2157.s1 Appendix. Figure A Testing for common trends across cohorts.
