# Supplementary material for: Medical Student Engagement and Intention to Practise Medicine Across Undergraduate Years: A Longitudinal Study
Source: Perspect Med Educ. 2026 Oct 6;15(1):1058–72. doi: 10.5334/pme.2157 (PMC13651775; doi:10.5334/pme.2157)
Supplement: Supplementary file 1 [file pme-15-1-2157-s1.pdf]

# Appendix

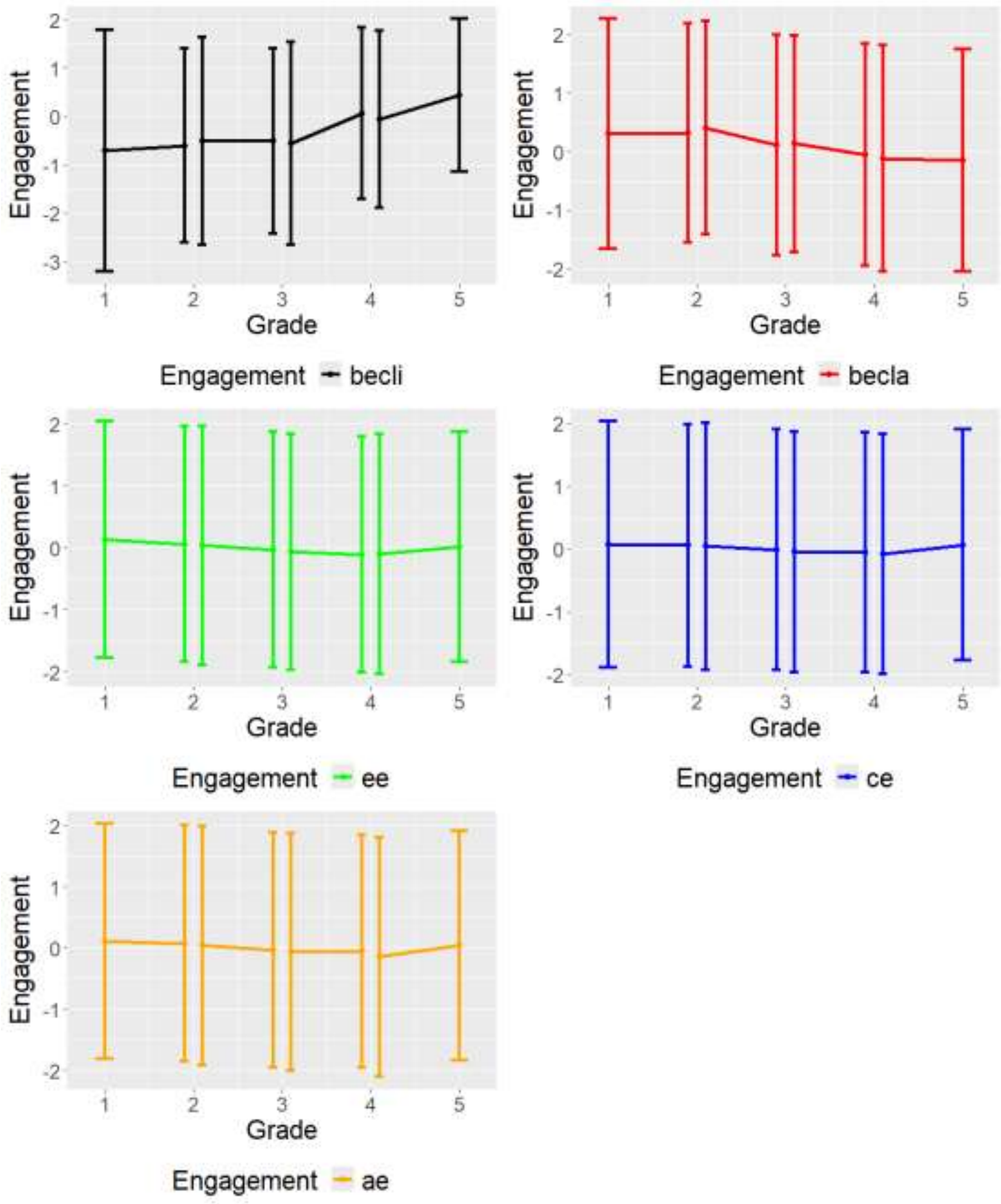


**Figure A** Testing for common trends across cohorts

Notes: The graph displays the results of testing for common trends across cohorts. Specifically, we present the mean and 95% confidence intervals of the factor scores derived from the factor analysis for each student engagement. It can be seen that adjacent cohort means are fairly similar, and the confidence intervals of the cohorts overlap. Therefore, it is reasonable to use all four cohorts to fit the complete undergraduate engagement curve at the same time period. The vertical axis represents the factor analysis scores for each engagement
